# Construction of Circular RNA–MicroRNA–Messenger RNA Regulatory Network of Recurrent Implantation Failure to Explore Its Potential Pathogenesis

**DOI:** 10.3389/fgene.2020.627459

**Published:** 2021-02-16

**Authors:** Jiahuan Luo, Li Zhu, Ning Zhou, Yuanyuan Zhang, Lirong Zhang, Ruopeng Zhang

**Affiliations:** ^1^Clinical Medical College, Dali University, Dali, China; ^2^Department of Reproductive Medicine, The First Affiliated Hospital of Dali University, Dali, China; ^3^Institute of Reproductive Medicine, Dali University, Dali, China

**Keywords:** recurrent implantation failure, circRNA, competitive endogenous RNA, GEO, network

## Abstract

**Background:** Many studies on circular RNAs (circRNAs) have recently been published. However, the function of circRNAs in recurrent implantation failure (RIF) is unknown and remains to be explored. This study aims to determine the regulatory mechanisms of circRNAs in RIF.

**Methods:** Microarray data of RIF circRNA (GSE147442), microRNA (miRNA; GSE71332), and messenger RNA (mRNA; GSE103465) were downloaded from the Gene Expression Omnibus (GEO) database to identify differentially expressed circRNA, miRNA, and mRNA. The circRNA–miRNA–mRNA network was constructed by Cytoscape 3.8.0 software, then the protein–protein interaction (PPI) network was constructed by STRING database, and the hub genes were identified by cytoHubba plug-in. The circRNA–miRNA–hub gene regulatory subnetwork was formed to understand the regulatory axis of hub genes in RIF. Finally, the Gene Ontology (GO) analysis and Kyoto Encyclopedia of Genes and Genomes (KEGG) pathway enrichment analysis of the hub genes were performed by clusterProfiler package of Rstudio software, and Reactome Functional Interaction (FI) plug-in was used for reactome analysis to comprehensively analyze the mechanism of hub genes in RIF.

**Results:** A total of eight upregulated differentially expressed circRNAs (DECs), five downregulated DECs, 56 downregulated differentially expressed miRNAs (DEmiRs), 104 upregulated DEmiRs, 429 upregulated differentially expressed genes (DEGs), and 1,067 downregulated DEGs were identified regarding RIF. The miRNA response elements of 13 DECs were then predicted. Seven overlapping miRNAs were obtained by intersecting the predicted miRNA and DEmiRs. Then, 56 overlapping mRNAs were obtained by intersecting the predicted target mRNAs of seven miRNAs with 1,496 DEGs. The circRNA–miRNA–mRNA network and PPI network were constructed through six circRNAs, seven miRNAs, and 56 mRNAs; and four hub genes (YWHAZ, JAK2, MYH9, and RAP2C) were identified. The circRNA–miRNA–hub gene regulatory subnetwork with nine regulatory axes was formed in RIF. Functional enrichment analysis and reactome analysis showed that these four hub genes were closely related to the biological functions and pathways of RIF.

**Conclusion:** The results of this study provide further understanding of the potential pathogenesis from the perspective of circRNA-related competitive endogenous RNA network in RIF.

## Introduction

Recurrent implantation failure (RIF) refers to infertility in patients younger than 40 years who undergo at least three *in vitro* fertilizations (IVFs) (including fresh embryo transfer and frozen–thawed embryo transfer) or intracytoplasmic sperm injection (ICSI) cycles and implantation of four or more high-quality embryos without embryo implantation or clinical pregnancy (Bashiri et al., 2018). Studies have shown that RIF accounts for about 10% of IVF–embryo transplantation (IVF-ET) (Simur et al., 2009). RIF causes serious mental stress and economic burden to families and even brings a lot of social problems. However, up to now, RIF is still an unsolved problem in assisted reproductive technology. The etiology of RIF has not been elucidated, and there is a lack of effective therapies and no reliable molecular markers to predict the occurrence of RIF. Therefore, elucidating the molecular mechanism of RIF is essential for the development of effective diagnostic and therapeutic targets.

Circular RNAs (circRNAs) are a type of non-coding RNAs that exist in almost all cells of an organism. The 3′ and 5′ ends of circRNA are covalently linked to form a closed circular single-stranded structure, which enables it to resist the hydrolysis of exonucleases and thus has relative stability and conservation (Shao et al., 2017; Shi et al., 2020). In addition, tissue-specific expression and rich diversity of circRNA have made it to be considered the best biomarkers (Chen et al., 2019), some of which have been identified as diagnostic and prognostic biomarkers. Recently, increasing evidence has shown that circRNAs are involved in various cellular processes such as gene expression regulation, cell cycle progression, and chromatin modification (Beermann et al., 2016; Wang Y. et al., 2018; Zang et al., 2020). In summary, the study of circRNAs has become a new hotspot in the field of RNA due to their various functions and specific properties.

Accumulating evidence suggests that circRNAs exert biological processes, including the genesis, translation, and transcriptional regulation of target genes, and extracellular transport, by acting as microRNA (miRNA) sponges, transcriptional activators or inhibitors, and RNA-binding protein (RBP) sponges (Zang et al., 2020). Some circRNAs can even encode polypeptides or proteins to participate in biological regulation (Li et al., 2017; Yang et al., 2017; Han et al., 2018; Xia et al., 2018). Recent studies show that circRNAs exert their functions mainly by adsorbing miRNAs to regulate miRNA expression, thereby regulating the target genes of miRNAs, of which circRNAs are called competing endogenous RNA (ceRNA). In the study of gynecologic tumors, it was found that the expression of circRNA not only promoted cancer but also inhibited cancer. In studies of cervical cancer, hsa_circRNA_101996 was highly expressed in cervical cancer cells. Hsa_circRNA_101996 regulates the proliferation, cell cycle, migration, and invasion of cervical cancer cells mainly through miR-8075 targeting TPX2 (Song et al., 2019), with higher levels of hsa_circRNA_101996 associated with a poor prognosis. Wang H. et al. (2018) found that circRNA-000911 expression was significantly downregulated in breast cancer cells. The high expression of circRNA-000911 could antagonize miR-449a, thereby increasing Notch1 expression to inhibit cell proliferation, migration, and invasion and to promote apoptosis of breast cancer cells. Lu H. et al. (2020) found that CIRS-126 regulated the expression of programmed cell death protein 4 (PDCD4) and inhibited the proliferation of ovarian granulosa cells by acting as a miR-21 sponge in polycystic ovary syndrome. In summary, circRNA–miRNA–messenger RNA (mRNA) regulatory network plays an important role in the occurrence and development of gynecologic diseases, while circRNA has different targets and functions in different tissue cells. Liu et al. (2017) performed microarray sequencing on endometrial biopsies from patients with RIF and found differentially expressed circRNAs (DECs). However, the specific targets and mechanisms of circRNA in RIF have not been reported.

**Figure 1:**
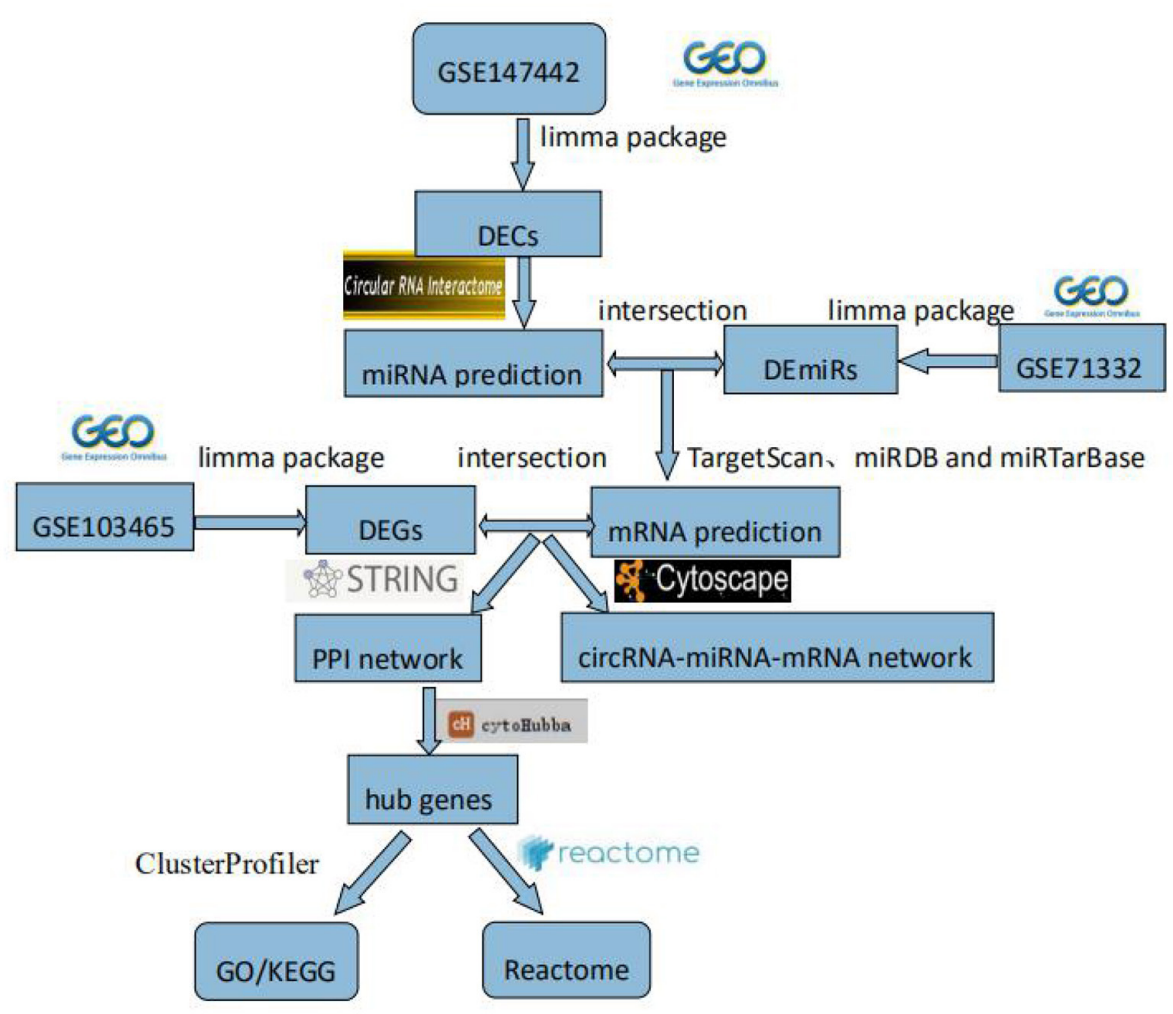
Flowchart. GEO, Gene Expression Omnibus; DECs, differentially expressed circular RNAs; DEmiRs, differentially expressed microRNAs; DEGs, differently expressed genes; PPI, protein–protein interaction; GO, Gene Ontology; KEGG, Kyoto Encyclopedia of Genes and Genomes.

In this study, we explored novel circRNAs and their mechanisms in the endometrium of patients with RIF through bioinformatics analysis. First, RIF-related circRNAs miRNA, and mRNA microarray data were collected from the Gene Expression Omnibus (GEO) database. DECs, differentially expressed miRNAs (DEmiRs), and differentially expressed genes (DEGs) were identified by RStudio software. The circRNA–miRNA–mRNA network was constructed by Cytoscape 3.8.0 software, and then protein–protein interaction (PPI) network was constructed by STRING (Search Tool for the Retrieval of Interacting Genes) (version 11.0) database, and hub genes were identified by cytoHubba plug-in. The circRNA–miRNA–hub gene regulatory subnetwork was formed to understand the regulatory axis of hub genes in RIF. Finally, in order to explore the potential role of hub genes in the development of RIF, Gene Ontology (GO), Kyoto Encyclopedia of Genes and Genomes (KEGG), and Reactome Functional Interaction (FI) enrichment analyses of hub genes were performed. This flowchart is shown in Figure 1.

## Materials and Methods

### Data Extraction

Microarray data of RIF circRNA, miRNA, and mRNA were downloaded from GEO database (http://www.ncbi.nlm.nih.gov/geo/) to identify DECs, DEmiRs, and DEGs. CircRNA expression data were derived from GSE147442 microarray (eight endometrial biopsy tissues from RIF patients and eight endometrial biopsy tissues from healthy controls). And GPL21825 074301 Arraystar Human CircRNA microarray V2 (Agilent Technologies, Inc., Palo Alto, CA) provided annotation information to convert probes into recognizable RNAs. Similarly, GSE71332 microarray and the corresponding GPL18402 Agilent-046064 Unrestricted_Human_miRNA_V19.0_ Microarray (miRNA ID version) were used to extract miRNAs, endometrial biopsy tissues from seven RIF patients and five normal pregnant women. Considering the type of specimen and the availability of data, GSE103465 and the corresponding GPL16043 GeneChip® PrimeView™ Human Gene Expression Array (with External spike-in RNAs) were used for the extraction of mRNA using a total of six samples, including three endometrial biopsies from RIF patients and three from pregnant women. For three microarrays, it can be seen that the difference of general data between the cases and controls is not statistically significant.

### Identification of Differentially Expressed Circular RNAs, Differentially Expressed MicroRNAs, and Differentially Expressed Messenger RNAs

Data were extracted and normalized by RStudio software, and then DECs, DEmiRs, and DEGs in the endometrium of RIF patients were obtained by limma package based on the Bioconductor package. The selection criteria for DECs were false discovery rate (FDR) < 0.05, |log_2_FC| > 2, for DEmiRs were FDR < 0.05, |log_2_FC| > 0.5, and for DEGs were FDR < 0.05. |log_2_FC| > 1 was considered to be a statistically significant difference.

### Prediction of Circular RNA–MicroRNA Pairs

CircRNAs act as sponges for miRNAs through the miRNA response elements (MREs). The Circular RNA Interactome online tool (https://circinteractome.nia.nih.gov/) was applied to predict target miRNAs of DECs of RIF. Overlapping miRNAs were obtained by intersecting predicted miRNAs and DEmiRs.

### Prediction Target Genes of MicroRNAs

The software TargetScan (http://www.targetscan.org/vert_72/), miRDB (http://www.mirdb.org/), and miRTarBase (http://mirtarbase.mbc.nctu.edu.tw/php/search.php) were used to predict the target genes of miRNA, and the intersection part were selected as the predicted mRNAs by Venn diagram in RStudio software. Overlapping mRNAs were obtained by intersecting predicted miRNAs and DEmiRs.

### Construction of Circular RNA–MicroRNA–Messenger RNA Network

The above differentially expressed circRNA–miRNA pairs and overlapping mRNAs were used to construct circRNA–miRNA–mRNA network, which were input into the Cytoscape 3.8.0 software program (https://cytoscape.org/) to visualize their circRNA-related ceRNA network.

### Construction of Protein–Protein Interaction Network and Identification of Hub Genes

In organisms, although there are multiple genes acting on the same trait, not all expressed genes play an equally important role, and a gene contributes greatly to a certain trait as hub gene. Finding the hub genes acting on RIF would help to understand the molecular mechanisms of this disease. First, a PPI network was built based on DEGs in circRNA–miRNA–mRNA network by STRING (Search Tool for the Retrieval of Interacting Genes) (v11.0) (https://string-db.org/cgi/input.pl) online software and was visualized by Cytoscape 3.8.0 software program. Then the degree, betweenness centrality, and closeness centrality of mRNAs in the PPI network were used to identify RIF-related hub genes by “cytoHubba” plug-in (Chin et al., 2014). We set “hubba nodes” for the top five nodes ranked by degree, closeness, and betweenness. Overlapping, top-ranking genes among the three algorithms were selected as hub genes.

### Gene Ontology and Kyoto Encyclopedia of Genes and Genomes Enrichment Analyses and Reactome Analysis of Hub Genes

GO analysis and KEGG pathway enrichment analyses of hub genes were performed by clusterProfiler package in RStudio software. Reactome pathway analysis was conducted with Reactome FI plug-in to comprehensively analyze the molecular mechanism of hub genes in RIF.

## Results

### Identification of Differentially Expressed Circular RNAs, Differentially Expressed MicroRNAs, and Differentially Expressed Messenger RNAs

**Figure 2:**
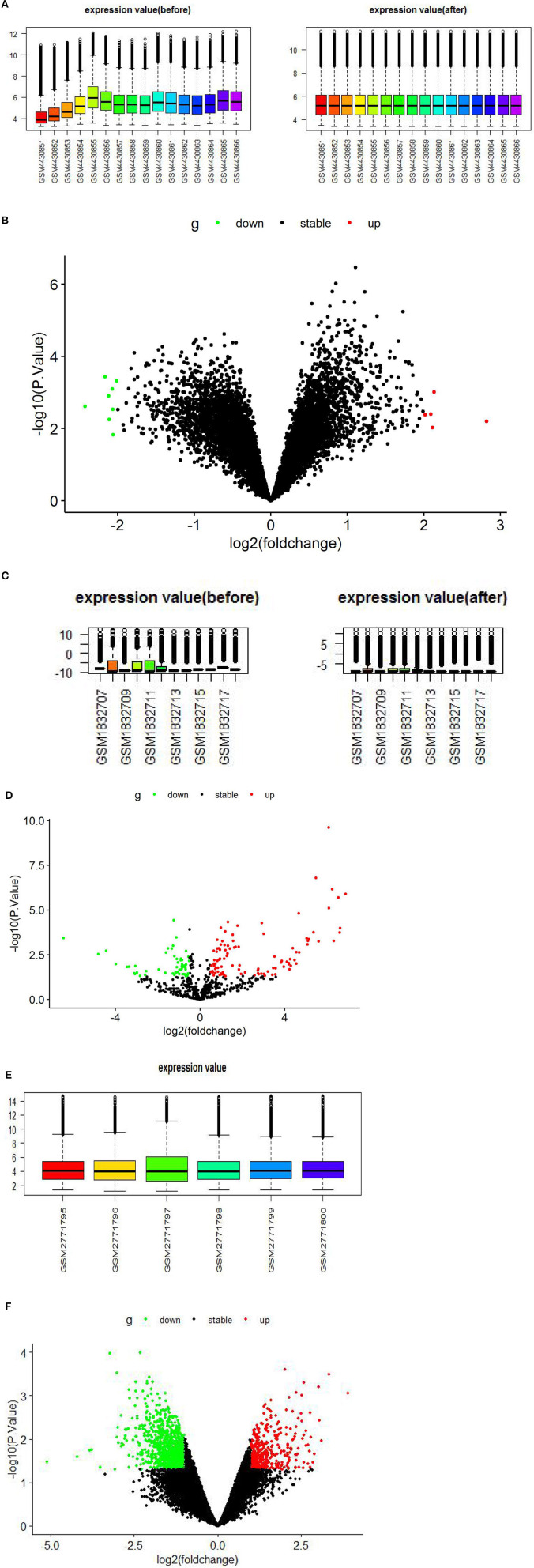
Boxplots and volcano plots for each microarray. **(A)** Boxplot of GSE147442 before and after standardization. **(B)** Volcano plots of DECs based on GSE147442. **(C)** Boxplot of GSE71332 before and after standardization. **(D)** Volcano plots of DEmiRs based on GSE71332. **(E)** Boxplot of GSE103465. **(F)** Volcano plots of DEGs based on GSE103465. DECs, differentially expressed circular RNAs; DEmiRs, differentially expressed microRNAs; DEGs, differently expressed genes.

The GSE147442 microarray was extracted and normalized by RStudio software, analyzed by the limma package in RStudio software, and identified 13 DECs, including eight downregulated DECs and five upregulated DECs (Figures 2A,B, Supplementary Table 1). A total of 160 DEmiRs were obtained in the GSE71332 microarray. Of these, 56 were downregulated and 104 upregulated DEmiRs (Figures 2C,D, Supplementary Table 2). Similarly, we performed the same analysis on GSE103465 microarray and found 1,559 DEGs, including 492 upregulated and 1,067 downregulated DEGs (Figures 2E,F, Supplementary Table 3).

### Prediction of Circular RNA–MicroRNA Pairs

The circRNA–miRNA pairs corresponding to 14 DECs were predicted by Circular RNA Interactome online software. The predicted miRNAs and 160 DEmiRs obtained from the microarray were intersected, and finally 11 circRNA–miRNA pairs were identified, including six circRNAs (hsa_circ_0058161, hsa_circ_0033392, hsa_circ_0030162, hsa_circ_0004121, hsa_circ_0034642, and hsa_circ_0034762) and seven miRNAs (hsa-miR-1290, hsa-miR-1305, hsa-miR-375, hsa-miR-370, hsa-miR-887, hsa-miR-1225-5p, and hsa-miR-1825).

### Prediction Target Genes of MicroRNAs

**Figure 3:**
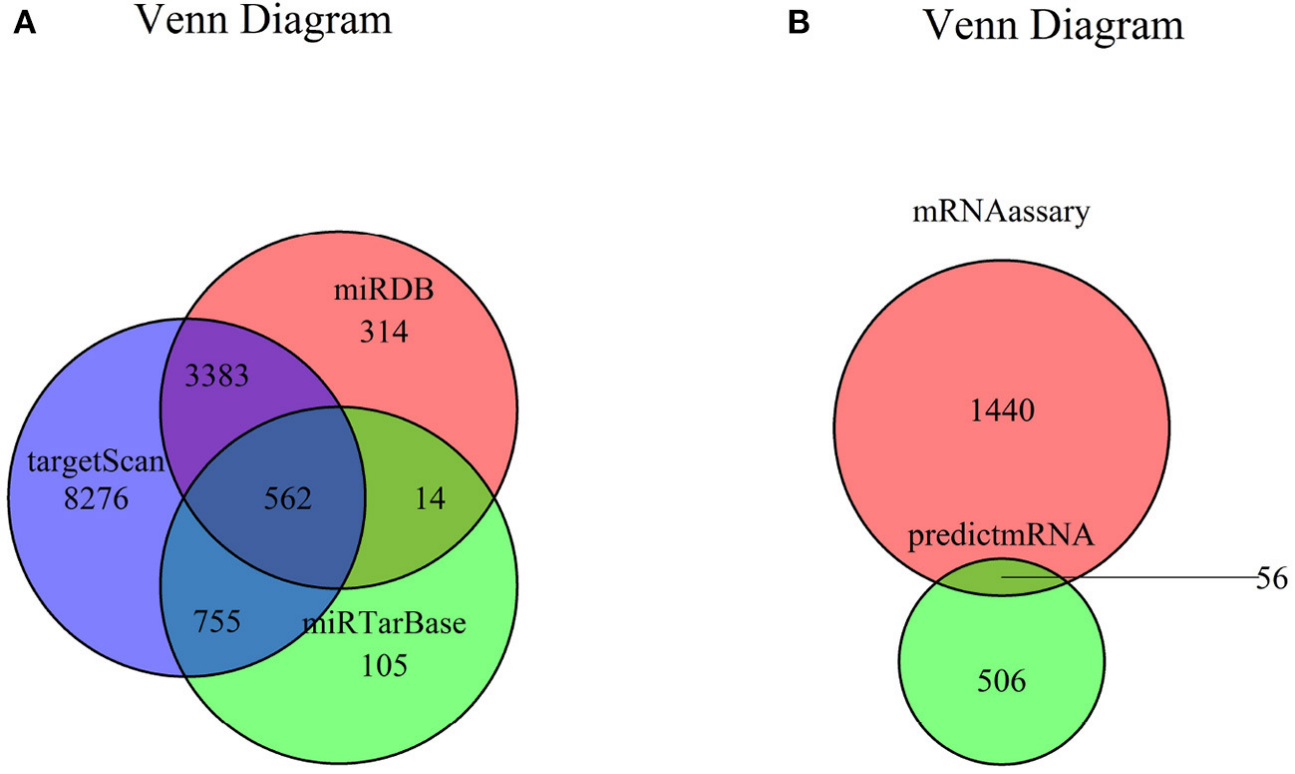
Venn diagram of mRNA. **(A)** Venn diagram of mRNA predicted by TargetScan, miRDB, and miRTarBase. **(B)** Venn diagram of DEGs and predicted mRNAs. mRNA, messenger RNA; DEG, differently expressed genes.

The target genes of seven miRNAs were predicted by TargetScan, miRDB, and miRTarBase software; and 562 intersected mRNAs were selected as predicted target genes by Venn diagram in RStudio (Figure 3A). The intersection of the predicted 562 miRNA target genes with 1,559 DEGs yielded 56 overlapping mRNAs (Figure 3B, Supplementary Table 4).

### Construction of Circular RNA–MicroRNA–Messenger RNA Network

**Figure 4:**
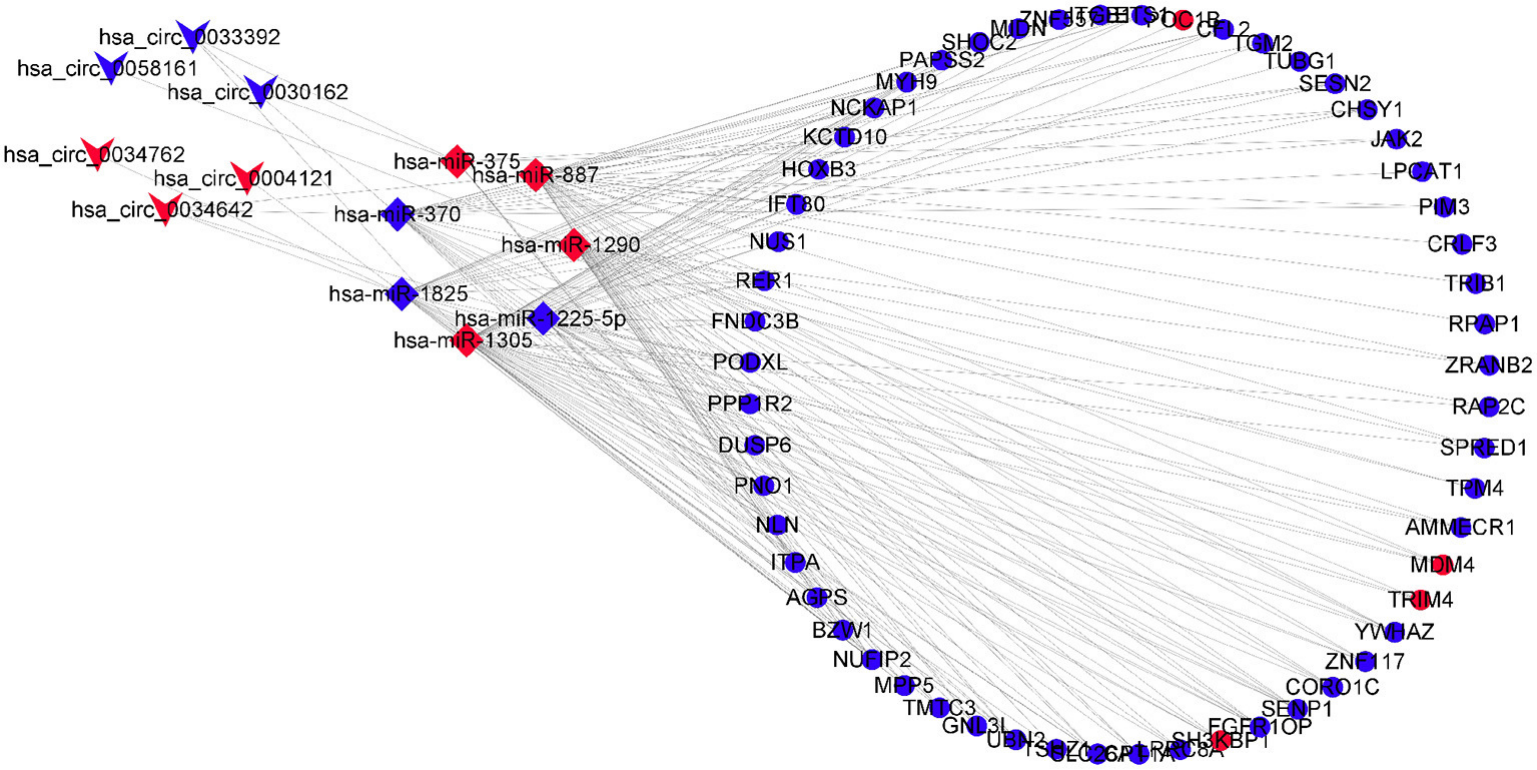
CircRNA–miRNA–mRNA regulatory network, which consists of six DECs, seven DEmiRs, and 56 DEGs. DECs, differentially expressed circular RNAs; DEmiRs, differentially expressed microRNAs; DEGs, differently expressed genes.

A circRNA–miRNA–mRNA network was constructed through six DECs, seven DEmiRs, and 56 DEGs and visualized by the Cytoscape 3.8.0 software program (Figure 4).

### Identification of Four Hub Genes in Protein–Protein Interaction Network by cytoHubba Plug-In

**Figure 5:**
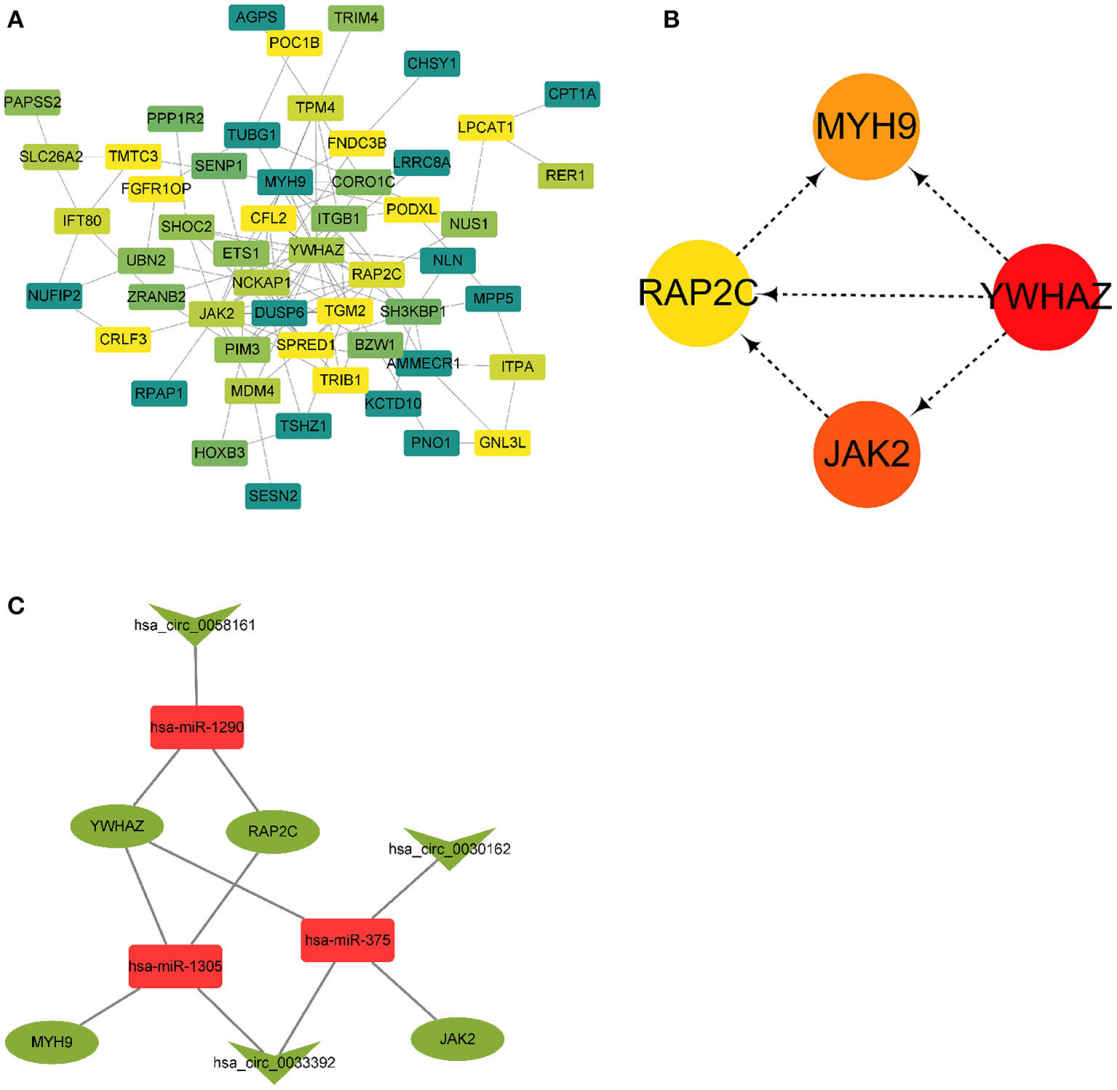
A PPI network and circRNA–miRNA–hub gene regulatory subnetwork. **(A)** A PPI network of the 56 target genes that exert important roles in RIF. This network consists of 53 nodes and 117 edges. **(B)** Four hub genes extracted by cytoHubba plug-in. **(C)** CircRNA–miRNA–hub gene regulatory subnetwork, consisting of three circRNAs, three miRNAs, and four mRNAs. PPI, protein–protein interaction; circRNA, circular RNA; miRNA, microRNA; RIF, recurrent implantation failure.

A PPI network based on 56 differentially expressed genes was constructed in circRNA–miRNA–mRNA network to understand the interaction of differentially expressed genes by STRING software, and it was visualized by Cytoscape 3.8.0 software program (Figure 5A), which contained 56 nodes and 117 edges. Then the top five genes obtained by the degree, betweenness centrality, and closeness centrality algorithms in cytoHubba plug-in are listed in Table 1; and overlapping genes were selected as hub genes YWHAZ, JAK2, MYH9, and RAP2C (Figure 5B). Then a circRNA–miRNA–hub gene subnetwork with nine regulatory modules, including hsa_circ_0058161/hsa-miR-1290/YWHAZ regulatory axis, hsa_circ_0058161/hsa-miR-1290/RAP2C regulatory axis, hsa_circ_0030162/hsa-miR-375/JAK2 regulatory axis, hsa_circ_0030162/hsa-miR-375/YWHAZ regulatory axis, hsa_circ_0033392/hsa-miR-375/JAK2 regulatory axis, hsa_circ_0033392/hsa-miR-375/YWHAZ regulatory axis, hsa_circ_0033392/hsa-miR-1305/YWHAZ regulatory axis, hsa_circ_0033392/hsa-miR-1305/MYH9 regulatory axis, and hsa_circ_0033392/hsa-miR-1305/RAP2C regulatory axis, was constructed to depict the relationship between circRNAs, miRNAs, and hub genes (Figure 5C).

**Table 1:** The top five genes obtained by the degree, betweenness centrality, and closeness centrality algorithms in cytoHubba plug-in.

**Name**	**Degree**	**Name**	**Betweenness**	**Name**	**Closeness**
YWHAZ	19	YWHAZ	800.89	YWHAZ	33.42
JAK2	15	MYH9	464.27	JAK2	30.45
MYH9	11	RAP2C	391.39	MYH9	28.50
RAP2C	10	JAK2	373.42	RAP2C	28.08
DUSP6	9	NUS1	296.07	CFL2	26.92

### Gene Ontology Annotation, Kyoto Encyclopedia of Genes and Genomes Pathway, and Reactome Pathway Analyses of Four Hub Genes

**Figure 6:**
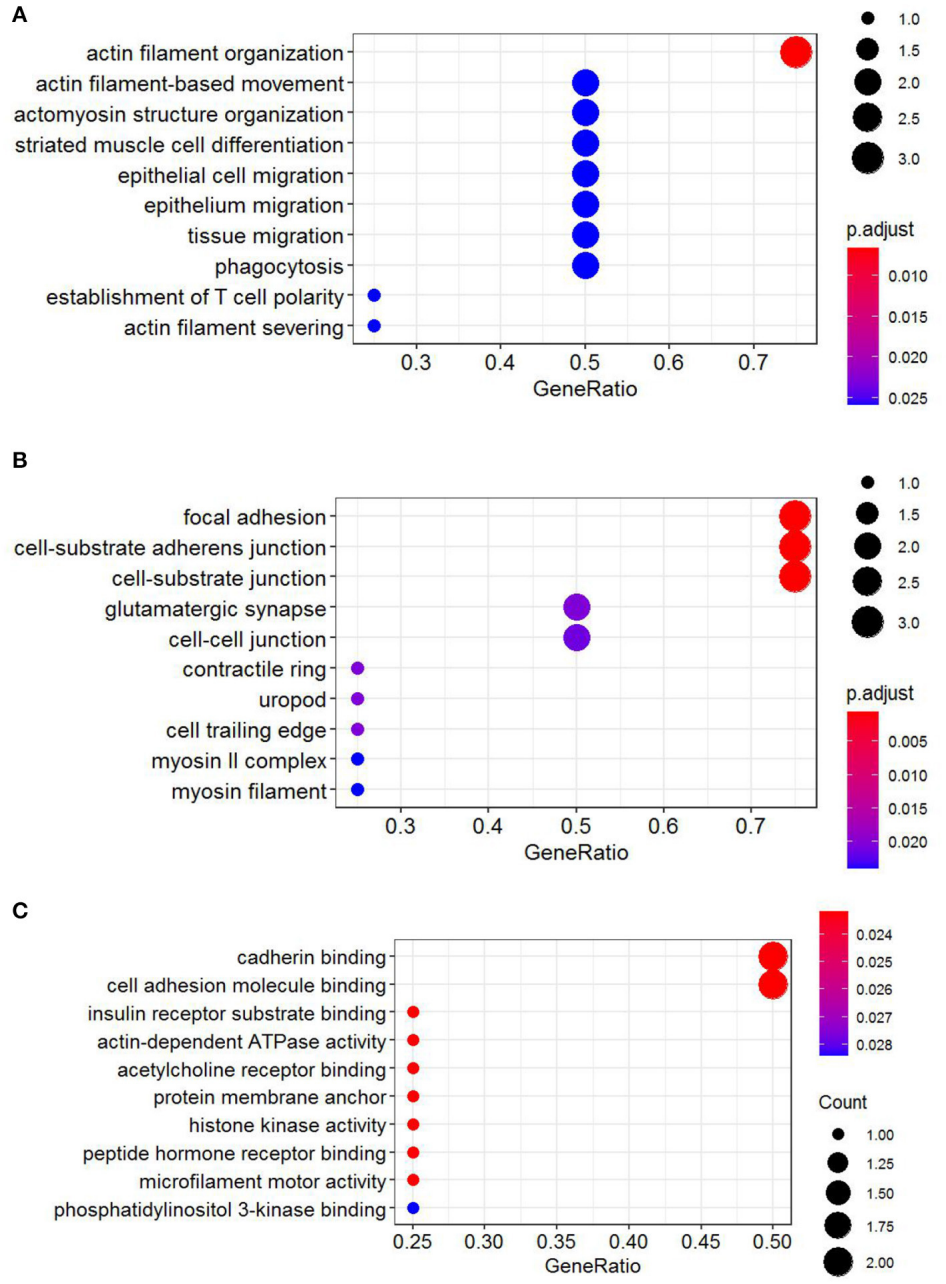
GO functional annotation of four hub genes. **(A)** Biological process (BP). **(B)** Cellular component (CC). **(C)** Molecular function (MF). GO analysis was conducted by R package “clusterProfiler” and visualized by R package “ggplot2.” GO, Gene Ontology.

Functional annotation of four hub genes was performed by GO analysis. GO terms for biological process (BP), cellular component (CC), and molecular function (MF) are shown in Figure 6. The most important GO terms were as follows: “actin filament organization” (*P* < 0.01) in BP, “focal adhesion” (*P* < 0.005) in CC, and “cadherin binding” (*P* < 0.024) in MF. KEGG pathway analysis was also performed to identify the signaling pathways involved in these four hub genes. Two significantly enriched pathways were found (*P* < 0.05) (Table 2), in which the “tight junction” was reported to be associated with the progression of RIF (Bellati et al., 2019). In addition, reactome pathway analysis of four hub genes is shown in Table 3.

**Table 2:** KEGG pathway analysis of four hub genes.

**ID**	**Description**	**GeneRatio**	**BgRatio**	**pvalue**	**p.adjust**	**qvalue**	**geneID**	**Count**
hsa04530	Tight junction	2/4	162/8,063	0.002	0.035	0.025	4,627/57,826	2
hsa05161	Hepatitis B	2/4	162/8,063	0.002	0.035	0.025	3,717/7,534	2

*Kyoto Encyclopedia of Genes and Genomes (KEGG) analysis was conducted by R package “clusterProfiler”*.

**Table 3:** Reactome pathway analysis of four hub genes.

**Reactome pathway**	***P*-value**	**FDR**	**HitGenes**
RHO GTPases activate PKNs	1.02E−04	4.53E−03	MYH9, YWHAZ
Interleukin-3, Interleukin-5 and GM-CSF signaling	1.13E−04	4.53E−03	JAK2, YWHAZ
Translocation of SLC2A4 (GLUT4) to the plasma membrane	2.03E−04	5.29E−03	MYH9, YWHAZ
Erythropoietin activates STAT5	3.21E−03	0.0155	JAK2
Erythropoietin activates Phospholipase C gamma (PLCG)	3.21E−03	0.0155	JAK2
MAPK1 (ERK2) activation	3.66E−03	0.0155	JAK2
RHO GTPase Effectors	3.92E−03	0.0155	MYH9, YWHAZ
MAPK3 (ERK1) activation	4.12E−03	0.0155	JAK2
Regulation of localization of FOXO transcription factors	4.12E−03	0.0155	YWHAZ
Interleukin-23 signaling	4.12E−03	0.0155	JAK2

*Reactome pathway analysis was conducted by Reactome FI plug-in*.

*FDR, false discovery rate*.

## Discussion

CircRNA is a stable non-coding RNA that has long been neglected by transcriptomics due to the lack of a 5′ cap and a 3′ polyadenylated tail. In the past decades, with the development of high-throughput sequencing and bioinformatics analysis, a large number of circRNAs have been unveiled in various tissues and cells (Chen and Yang, 2015). Accumulating studies have revealed the important role of circRNAs in a variety of human diseases (Hu et al., 2018; Li et al., 2018; Yang et al., 2018). Because circRNAs exhibit specific expression in tissues or developmental stages, the function of circRNAs is still not fully understood (Hu et al., 2018; Li et al., 2018; Yang et al., 2018). Compared with linear RNAs, the higher stability of circRNAs conferred by their circular structure makes these circRNAs potentially valuable as important transcriptional regulators (Meng et al., 2017; Jiang et al., 2018). CircRNAs are commonly used as diagnostic and prognostic biomarkers. However, the exact role of circRNAs in RIF remains largely unknown. To determine whether circRNAs play a role in RIF, we first performed GEO microarray dataset selection and identified 13 DECs.

Current evidence suggests that circRNAs contain multiple MREs that can bind to miRNAs, commonly called “miRNA sponges,” which relieve the targeted inhibition of downstream mRNAs by miRNAs (Jin et al., 2019; Lu J. et al., 2020; Qiu et al., 2020), thereby regulating the expression of protein-coding genes. In this study, in order to investigate whether the 13 DECs play a role in RIF as ceRNAs, their related MREs were predicted by Circular RNA Interactome software. The predicted target miRNAs were interacted with DEmiRs from GEO miRNA microarrays, and overlapping miRNAs were taken for further study. Ultimately, 11 circRNA–miRNA pairs were obtained, including six circRNAs (hsa_circ_0058161, hsa_circ_0033392, hsa_circ_0030162, hsa_circ_0004121, hsa_circ_0034642, and hsa_circ_0034762) and seven miRNAs (hsa-miR-1290, hsa-miR-1305, hsa-miR-375, hsa-miR-370, hsa-miR-887, hsa-miR-1225-5p, and hsa-miR-1825). After interacting 562 miRNA-related target genes and 1,559 DEGs, 56 overlapping genes were obtained to construct a circRNA-related ceRNA regulatory network. To further identify the key circRNAs involved in the regulatory network, we constructed a PPI network to screen the hub genes. Four hub genes (YWHAZ, JAK2, MYH9, and RAP2C) were identified. Functional annotation and pathway analysis indicated that the four hub genes were involved in multiple cellular functions and signaling pathways in RIF, including “actin filament Organization,” “tight junction,” and “RHO GTPases activate PKNs.”

To investigate the role of circRNA in RIF, a circRNA–miRNA–hub gene regulatory network was constructed based on the circRNA–miRNA–mRNA regulatory network. Hsa_circ_0058161, hsa_circ_0033392, and hsa_circ_0030162 were identified as the key circRNA in this network. GO enrichment analysis showed that the genes in this network were mainly involved in the regulation of actin filament organization, focal adhesion, and cadherin binding. Embryo implantation involves the adhesion of trophoblast cells to the epithelial layer of the endometrium, dependent on cell–cell adhesion molecule interactions (Heneweer et al., 2002). Relevant studies found that the expression of adhesion molecules β-catenin, E-cadherin, and K-cadherin in the endometrium of infertile patients was significantly lower than that of fertile patients, while the expression of β-catenin and E-cadherin was higher at the glandular level than in fertile patients (Koler et al., 2009). However, K-catenin and E-cadherin were lower in glandular levels with recurrent pregnancy loss than fertile patients, suggesting that cadherin is associated with endometrial receptivity and glands (Koler et al., 2009). It is speculated that hub genes affect RIF mainly by acting on the endometrium and related glands at the attachment of embryos through adhesion. KEGG pathway analysis found that hub genes were involved in the development of RIF through the tight junction pathway, which is the part of the interconnection network of adhesion complexes, which generate crosstalk through direct PPIs and interactions affecting their assembly and functional signaling. Karakotchian and Fraser (2007) showed that tight junctions play an important role in the process of embryo implantation, which is consistent with the results of this study. Reactome analysis revealed that MYH9 and YWHAZ could participate in the occurrence of RIF through RHO GTPases activate PKNs. RHO GTPases are important signal transduction molecules involved in a variety of important cell activities, such as actin cytoskeleton remodeling, cell movement, cell adhesion, gene expression, and cell cycle regulation (Bora and Shrivastava, 2017). Heneweer et al. (2002) measured the adhesion of RL95-2 cells of the uterine epithelium to JAR spheres by centrifugal force-based adhesion assay, and they found that the adhesion force depends on RHO GTPases, suggesting that RHO GTPases are most likely to play an important role in the binding of RL95-2 cells to trophoblast in the uterine epithelium. It is speculated that RHO GTPases activate PKNs that mainly affect the adhesion between the endometrial epithelium and gestational trophoblast in this study. These results indirectly suggest that circRNAs in this network may play a key role in the occurrence and development of RIF. This result deserves further study.

YWHAZ, also known as tyrosine 3 monooxygenase/tryptophan 5-monooxygenase activation protein zeta (14-3-3ζ), is a hub gene of many signal transduction pathways and plays a key role in the progression of multiple diseases (Wang et al., 2017; Yang et al., 2019; Gan et al., 2020). More and more studies have shown that YWHAZ is upregulated in breast cancer, ovarian cancer, G2 endometrial adenocarcinoma, prostate cancer, and other types of genitourinary tumors and that it participates in cell growth, cell cycle, apoptosis, migration, and invasion (Jeda et al., 2014; Wang et al., 2017; Yang et al., 2019; Yu et al., 2020). Some studies in placenta and endometrial tissues have considered YWHAZ as a housekeeping gene (Meller et al., 2005; Vestergaard et al., 2011; Sadek et al., 2012a,b; Jeda et al., 2014; Nelissen et al., 2014; Li et al., 2016; Wang et al., 2017; Yang et al., 2019; Yu et al., 2020), and others have found that the expression of YWHAZ is high in the eutopic endometrium of baboons with endometriosis, contributing to the pathophysiology of endometriosis (Joshi et al., 2015). In 12Z cells (immortalized human endometrium), low expression of YWHAZ was also found in ectopic epithelial cell lines, resulting in reducing cell proliferation (Joshi et al., 2015), consistent with the findings of Li et al. (2019), while in our study, YWHAZ expression was found to be downregulated in endometrial tissues of RIF patients, possibly associated with reduced cell proliferation. However, the current research of YWHAZ in RIF is insufficient, so more studies are need for confirmation.

JAK2 links to the intracellular domain of many cytokine receptors for signal transduction. When cytokines bind to JAK2 receptors, the phosphorylation of JAK2 leads to the phosphorylation of other intracellular molecules, mainly through the JAK2–STATA3 pathway, which ultimately leads to gene transcription (Roskoski, 2016; Choy, 2019). It plays an important role in cytokine signal transduction and regulation of cell growth and gene expression. JAK2 inhibitors cooperate with SMO inhibitors to inhibit the growth and metastasis of breast cancer cells (Doheny et al., 2020). Ito et al. (2004) detected the expression of JAK2 in mouse embryos to understand the role of JAK2 in the regulation of early preimplantation development by reverse transcription–polymerase chain reaction analysis and immunocytochemistry and found that JAK2 was mainly localized in single-cell embryos. In the unfertilized oocytes and M-stage single-cell embryos, JAK2 localized on chromosomes. Xu et al. (2017) showed that JAK2-mediated sodium/hydrogen exchange activation regulated acute cell volume changes in the late single-cell stage of mouse preimplantation embryos. Dysregulation of cell volume in early preimplantation embryos may lead to embryonic development arrest. In this study, JAK2 expression reduced, presumably reducing sodium/hydrogen exchange activation leading to dysregulation of cell volume, which affected embryonic development and embryonic adhesion.

The non-myosin heavy chain nine gene (MYH9) is located on chromosome 22q12.3 and encodes a cytoskeletal contractile protein, non-smooth muscle myosin heavy chain IIA (Pecci et al., 2018). Kadam et al. (2006) found that MYH9 protein on gametes interacts with the non-glycosylated N-terminal conserved region of tubal glycoprotein, and one tubal glycoprotein can bind to two gametes, which is associated with capacitated sperm, oocytes, and developing embryos. Lamy et al. (2018) performed proteomic identification in fallopian tube fluid after ovulation and found that MHY9 could regulate sperm function. However, there are few studies on the expression and mechanism of MYH9 in the endometrium of patients with RIF, and more researches are needed to verify it.

RAP2C is a member of the Rap family of small GTP-binding proteins, and a study showed that RAP2C is mainly expressed in the liver, skeletal muscle, prostate, uterus, rectum, stomach, and bladder. The protein is located in the cytoplasm and is involved in regulating cell growth, differentiation, and apoptosis (Guo et al., 2007). RAP2C has been found to be an important molecular switch in the mitogen-activated protein kinase (MAPK) signaling pathway in breast cancer; RAP2C reduces apoptosis and promotes proliferation and migration through the MAPK signaling pathway (Zhu et al., 2020). Zhang et al. (2017) conducted a genome-wide associated study of 43,568 women of European descent and found that variations in the RAP2C locus were associated with duration of pregnancy; and the established roles of these genes in uterine development, maternal nutrition, and vascular control supported their mechanism involvement. Although RAP2C expresses in the uterus, the effect of changes in RAP2C expression on endometrial receptivity and RIF needs further study.

Nine circRNA–miRNA–hub gene regulatory modules, including hsa_circ_0058161/hsa-miR-1290/YWHAZ regulatory axis, hsa_circ_0058161/hsa-miR-1290/RAP2C regulatory axis, hsa_circ_0030162/hsa-miR-375/JAK2 regulatory axis, hsa_circ_0030162/hsa-miR-375/YWHAZ regulatory axis, hsa_circ_0033392/hsa-miR-375/JAK2 regulatory axis, hsa_circ_0033392/hsa-miR-375/YWHAZ regulatory axis, hsa_circ_0033392/hsa-miR-1305/YWHAZ regulatory axis, hsa_circ_0033392/hsa-miR-1305/MYH9 regulatory axis, and hsa_circ_0033392/hsa-miR-1305/RAP2C regulatory axis, were obtained from the final circRNA-related subnetwork. Overall, for four genes, hsa_circ_0033392 and hsa_circ_0030162 had a competitive regulatory relationship. However, so far, there is no research about hsa_circ_0058161, hsa_circ_0033392, and hsa_circ_0030162 on diseases published.

Hsa-miR-1290 overexpression was found in breast cancer (Hamam et al., 2016), glioblastoma (Khalighfard et al., 2020), and fatty liver disease (Tan et al., 2014). Consistent with this study, hsa-miR-1290 is a risk factor for RIF. As the downstream target genes of hsa-miR-1290 in this study, YWHAZ and RAP2C are associated with endometrial cell proliferation. It is speculated that hsa-miR-1290 induces endometrial cell proliferation inhibition and endometrial receptivity impairment leading to RIF by decreasing YWHAZ expression. However, hsa_circ_0058161/hsa-miR-1290/ YWHAZ axis has not been reported in the occurrence and development of RIF. The mechanism of RAP2C in RIF is not clear, so the mechanism of hsa_circ_0058161/hsa-miR-1290/RAP2C axis in RIF cannot be speculated.

Hsa-miR-375 gene is located in the intergenic region between beta-A2 crystallin (cryba2) and coiled-coil domain-containing protein 108 (ccdc108) genes in human chromosome 2q35 region, and the sequence of hsa-miR-375 is highly conserved (Baroukh and van Obberghen, 2009). Further studies have shown that hsa-miR-375 is a multifunctional miRNA involved in islet development, glucose homeostasis, mucosal immunity, pulmonary surfactant secretion, and tumorigenesis (Shao et al., 2014; Yan et al., 2014). In this study, we found that hsa-miR-375 is upregulated in RIF. However, there are few reports on the function of hsa-miR-375 in RIF or its interaction with upstream circRNA. Therefore, more research is necessary.

It has been found in cervical cancer that hsa-miR-1305 regulates the Wnt/β-catenin pathway by binding to Wnt2 to promote cell proliferation, migration, and invasion (Liu et al., 2020). Testing of peripheral blood samples of monozygotic discordant twins for epithelial ovarian carcinoma found that the expression of hsa-miR-1305 was upregulated and that hsa-miR-1305 regulates cell cycle and cell apoptosis (Tuncer et al., 2020). The expression of hsa-miR-1305 is rapidly upregulated after the initiation of pluripotent stem cell differentiation (within 24 h), indicating that it plays a role in early differentiation (Jin et al., 2016). Furthermore, the downregulation of hsa-miR-1035 contributes to the consolidation of the pluripotent phenotype, and its overexpression leads to the initiation of differentiation, thus suggesting that hsa-miR-1305 acts as a regulator to maintain a fine balance between pluripotency and differentiation. Overexpression of hsa-miR-1305 increases cell apoptosis, while its knockdown reduces the number of apoptotic cells (Jin et al., 2016). In this study, miR-1305 was found to be upregulated in RIF. However, there is no report linking miR1305 to RIF or its association with upstream circRNA.

At present, there are a few studies on the mechanism of circRNA in RIF. The novelty of this study is that the circRNA–miRNA–mRNA network was constructed for the first time through the GEO database. However, given that these results are only based on bioinformatics models, further in-depth research is crucial to verify the possible role of these nine axes in RIF.

## Conclusion

DECs, DEmiRs, and DEGs were identified from publicly available microarray data to construct circRNA-related ceRNA networks. The circRNA–miRNA–hub gene regulatory subnetwork reveals that three important circRNAs and four hub genes may be involved in the development of RIF, provides new insights into the pathogenesis of RIF, and proposes potential therapeutic targets worthy of further study.

## Data Availability Statement

The original contributions presented in the study are included in the article/Supplementary Material, further inquiries can be directed to the corresponding author/s.

## Author Contributions

JL and LZ: conceptualization. NZ and YZ: investigation and validation. LZ and RZ: methodology. JL: software. LZ: supervision. JL and LZ: writing—original draft preparation. LZ and RZ: writing—review and editing. All authors read and approved the final manuscript.

## Conflict of Interest

The authors declare that the research was conducted in the absence of any commercial or financial relationships that could be construed as a potential conflict of interest.

## Supplementary Material

Supplementary Table 1 13 DECs were obtained GSE147442 microarray by the limma package in RStudio software. DECs differentially expressed circRNAs.

Supplementary Table 2 160 DEmiRs were obtained GSE71332 microarray by the limma package in RStudio software. DEmiRs differentially expressed miRNAs.

Supplementary Table 3 1559 DEGs were obtained GSE103465 microarray by the limma package in RStudio software. DEGs differently expressed genes.

Supplementary Table 4 56 overlapping mRNAs were obtained by Venn diagram in RStudio software.

## Abbreviations

circRNA: circular RNA
miRNA: microRNA
ceRNA: competing endogenous RNA
DECs: differentially expressed circRNAs
DEmiRs: differentially expressed miRNAs
DEGs: differentially expressed genes (mRNAs)
IVF-ET: *in vitro* fertilization–embryo transplantation
ICSI: intracytoplasmic sperm injection
FDR: false discovery rate
GEO: Gene Expression Omnibus
GO: Gene Ontology
KEGG: Kyoto Encyclopedia of Genes and Genomes
PPI: protein–protein interaction
RIF: recurrent implantation failure
RBP: RNA-binding protein
PDCD4: programmed cell death protein 4
STRING: Search Tool for the Retrieval of Interacting Gene
MAPK: mitogen-activated protein kinase
cryba2: beta-A2 crystallin
ccdc108: coiled-coil domain-containing protein 108.
